# Evidence-Based Decision Support for a Structured Care Program on Polypharmacy in Multimorbidity: A Guideline Upgrade Based on a Realist Synthesis

**DOI:** 10.3390/jpm12010069

**Published:** 2022-01-07

**Authors:** Truc Sophia Dinh, Maria-Sophie Brueckle, Ana Isabel González-González, Joachim Fessler, Ursula Marschall, Manfred Schubert-Zsilavesz, Ferdinand M. Gerlach, Sebastian Harder, Marjan van den Akker, Ingrid Schubert, Christiane Muth, the EVITA Study Group

**Affiliations:** 1 Institute of General Practice, Goethe-University Frankfurt, Theodor-Stern-Kai 7, 60590 Frankfurt am Main, Germany; brueckle@allgemeinmedizin.uni-frankfurt.de (M.-S.B.); gonzalezgonzalez@allgemeinmedizin.uni-frankfurt.de (A.I.G.-G.); gerlach@allgemeinmedizin.uni-frankfurt.de (F.M.G.); m.vandenakker@allgemeinmedizin.uni-frankfurt.de (M.v.d.A.); muth@allgemeinmedizin.uni-frankfurt.de (C.M.); 2 Gemeinschaftspraxis für Allgemeinmedizin, 65439 Floersheim, Germany; joachim.fessler@t-online.de; 3 Department Medicine/Health Care Research, Barmer, Lichtscheider Str. 89, 42285 Wuppertal, Germany; ursula.marschall@barmer.de; 4 Institute of Pharmaceutical Chemistry/ZAFES, Goethe University, Max-von-Laue-Str. 9, 60438 Frankfurt am Main, Germany; schubert-zsilavecz@pharmchem.uni-frankfurt.de; 5 Institute for Clinical Pharmacology, Goethe-University Frankfurt, Theodor-Stern-Kai 7, 60590 Frankfurt am Main, Germany; harder@em.uni-frankfurt.de; 6 Department of Familiy Medicine, Maastricht University, P.O. Box 616, 6200 MD Maastricht, The Netherlands; 7 Academic Centre of General Practice, KU Leuven, Kapucijnenvoer 30, Blok J, 3000 Leuven, Belgium; 8 PMV Research Group, Faculty of Medicine and University Hospital Cologne, University of Cologne, Herderstrasse 52, 50931 Cologne, Germany; ingrid.schubert@uk-koeln.de; 9 Department of General Practice and Family Medicine, Medical Faculty East-Westphalia, University of Bielefeld, Universitaetsstrasse 25, 33615 Bielefeld, Germany

**Keywords:** polypharmacy, multimorbidity, evidence-based guideline, realist synthesis, medication management, patient centered care, continuity of care, elderly, stakeholder analysis

## Abstract

Evidence-based clinical guidelines generally consider single conditions, and rarely multimorbidity. We developed an evidence-based guideline for a structured care program to manage polypharmacy in multimorbidity by using a realist synthesis to update the German polypharmacy guideline including the following five methods: formal prioritization in focus groups; systematic guideline review of evidence-based multimorbidity/polypharmacy guidelines; evidence search/synthesis and recommendation development; multidisciplinary consent of recommendations; feasibility test of updated guideline. We identified the need for a better description of the target group, decision support, prioritization of medication, consideration of patient preferences and anticholinergic properties, and of healthcare interfaces. We conducted a systematic guideline review of eight guidelines and extracted and synthesized recommendations using the Ariadne principles. We also included 48 systematic reviews. We formulated and agreed upon 34 recommendations for the revised guideline. During the feasibility test, guideline use enabled 57% of GPs to identify problems, leading to medication changes in 49% and self-assessed improvement in 56% of patients. Although 58% of GPs felt that it was too long, 92% recommended it. Polypharmacy should be systematically reviewed at least annually. Patients, family members, and healthcare professionals should monitor and adjust it using prospective process validation, taking into account patient preferences and agreed treatment goals.

## 1. Introduction

Multimorbidity, defined as the presence of two or more simultaneous chronic health conditions in an individual, is becoming the norm in general practice [1]. Multimorbidity leads to polypharmacy, non-adherence to medication regimens, increased use of potentially inappropriate medications [2], omissions [3], high utilization of healthcare resources use and increased cost [4].

In order to improve the care of patients with chronic diseases, health care systems worldwide have developed and implemented so-called disease management programs (DMPs). In Germany, DMPs are accessible for patients insured by the statutory health insurance. DMPs are available for chronic diseases such as asthma, COPD or diabetes and aim to improve patients’ quality of life as well as the medical care of patients as a whole [5]. DMPs are based on evidence-based clinical guidelines. Evidence-based clinical guidelines have been introduced for chronic conditions to set standards of care and reduce variation in treatments. However, these guidelines generally consider only single conditions and rarely take into account the co-occurrence of multiple chronic conditions [6]. As a consequence, existing DMPs mainly focus on the single-disease paradigm and are therefore not suitable for patients with polypharmacy and multimorbidity.

A rising awareness of polypharmacy in general practice was the reason behind the development of the first German evidence-based guideline on polypharmacy, completed in 2013. The current paper describes how we systematically updated the evidence for this guideline based on a realist synthesis. Our objective was to develop a guideline for use in a polypharmacy management program and to take into account user and healthcare priorities, formulate recommendations for consensus with multiple stakeholders, and test its feasibility prior to authorization. By doing so, we expected to increase feasibility and user acceptance.

This project is part of the EVITA project (Evidence-based Polypharmacy Program with Implementation in Health Care). The aim of this guideline update was to provide evidence-based decision support for a structured care program for polypharmacy in multimorbidity.

## 2. Materials and Methods

To systematically update the existing guideline on polypharmacy, we performed a realist synthesis. Realist synthesis has proved themselves as a suitable approach for synthesizing evidence because it also considers why, how and under what conditions interventions work or do not work [7,8]. The process of a realist synthesis is comparable to systematic review and is further characterized by a strong focus on understanding the underlying causal mechanisms and a continuous involvement of stakeholders in all phases [8,9]. It allows implementation researchers to systematically and transparently synthesize relevant evidence, to account for contextual factors, and to examine mechanisms of action in a particular context [8].

A realist synthesis consists of four stages:

1. Defining the scope of the review;
2. Searching and appraising the evidence;
3. Extracting and synthesizing findings;
4. Drawing conclusions and making recommendations [8,10].

The methods used for the planned guideline update are described in Table 1.

The following stakeholders were included in multiple stages of the realist synthesis: The Leitliniengruppe Hessen (LLGH, Guideline Group of General Practitioners in Hesse), which consists of general practitioners who are especially trained in pharmacotherapy and quality circle work. They have developed several GP guidelines since 1998 and were deeply consulted in the following key stages of the synthesis: definition of scope, discussion and appraisal of evidence, formulation of recommendations, discussion and interpretation of practice test results.

In addition, 18 international and multidisciplinary experts (geriatrics, primary care, public health and health services research, epidemiology and pharmacy/pharmacology) were involved in the discussion and appraisal of evidence. These experts participated in a workshop, which was part of a 2-day symposium [12].

The developed recommendations were consented among the LLGH and authorized experts/representatives from other relevant medical disciplines/societies (AkdÄ—Drug Commission of the German Medical Association; AMK—Drug Commission of the German Pharmacist Association; DDG—German Diabetes Association, DEGAM—German College of General Practitioners and Family Physicians; DGfN—German Society of Nephrology; DGG—German Geriatric Society; DGGG—German Society of Gerontology and Geriatrics, DGIM—German College of Internal Medicine; DGK—German Cardiac Society; DGPPN—German Association for Psychiatry, Psychotherapy and Psychosomatics; German Pain Association; GAA—Society for Drug Utilisation Research and Pharmacoepidemiology; LLGH—Guideline Group of General Practitioners in Hesse; patient representative of the Federal Joint Committee; care expert).

## 3. Results

### 3.1. Define the Scope of the Review

#### Focus Groups with General Practitioners

To define the scope of the planned guideline update, we first carried out two focus group discussions with 30 general practitioners (GPs). Our aim was to identify key attributes for the updated guideline, the overall need for change, and further topics that should be considered in evidence searches. The focus groups were moderated by a senior researcher (C.M.), and notes from the discussions were taken and analyzed by the senior researcher (C.M.) and two research fellows (T.S.D. and B.F.). After completion of the analyses, the results were presented to and discussed with the LLGH (moderated by C.M. and I.S.), and topics for possible inclusion in the planned guideline update were formally prioritized.

During the focus groups, we recognized that the guideline structure would have to be improved in order to support its acceptance and implementation. To address topics of interest identified in the groups, we also decided to conduct a systematic review of evidence-based guidelines on multimorbidity and polypharmacy (see Section 3.2.1), as well as evidence searches based on systematic reviews (see Section 3.2.2). The topics of interest were as follows:

- Identification of the target group;
- Decision support in de-prescribing; prioritization of medication considering patient preferences and anticholinergic properties;
- Medication plan and monitoring;
- Healthcare interfaces.

Other key questions concerned how to collect information on patients’ medication, how to recognize medication-related risks, how to monitor symptoms, and how to avoid inappropriate medications. It was further decided not only to update but also to upgrade the existing guideline on polypharmacy from level S2e to level S3 (S2e: evidence-based guideline (incl. systematic searches, selection and appraisal of evidence); S3: evidence-based and expert-consented consented guideline (incl. representative committee, systematic searches, selection and appraisal of the evidence, and structured consensus process)) [13].

### 3.2. Search for and Appraise the Evidence

#### 3.2.1. Systematic Guideline Review

We conducted a systematic guideline review (SGR) [12] with the aim to identify, and systematically analyze recommendations from international evidence-based guidelines on polypharmacy and multimorbidity, and to describe their key concepts. Although the rationale behind the SGR and the results are described in detail elsewhere [12], we provide a brief summary here.

Electronic databases were searched (MEDLINE, The Cochrane Library, Health Services/Health Technology Assessment Texts (HSTAT), Turning Research Into Practice (TRIP), Guideline International Network (G-I-N), The National Guideline Clearinghouse) using controlled terms (‘polypharmacy’ OR ‘multiple drug’ OR ‘multimedication’ OR ‘multimorbidity’ OR ‘multiple conditions’, OR ‘comorbidity’). We did not apply any restrictions to the definition of multimorbidity or polypharmacy used in the guidelines, the publication date or to language. Guidelines or guideline-like documents were included when they contained explicit and systematically developed recommendations and had been endorsed by an official organization. Disease-specific guidelines and those with an overly narrow focus were excluded. Two independent researchers conducted the searches and selected guidelines for inclusion (T.S.D. and A.I.G.G.).

Three researchers (M.-S.B., T.S.D., A.I.G.G.) screened the titles and abstracts of 3939 records found in the databases and on the websites of guideline issuing organizations. Sixty-three guidelines underwent full-text screening. In total, we included four international guidelines on multimorbidity [14,15,16,17] and four on polypharmacy [18,19,20,21].

Four researchers (M.-S.B., J.W.B., T.S.D., A.I.G.G.) used the MiChe-Checklist to assess the quality of the guidelines [22,23]. Overall, the included guidelines were of good to very good methodological quality. Few had deficiencies, e.g., related to limited reporting on updates [12].

#### 3.2.2. Evidence Searches

We searched for further evidence to help answer specific research questions identified in the focus groups and revised the medication process as the central framework of the guideline. In the search, we used forward citation tracking (PUBMED: ‘similar articles’) and selected the Cochrane Review on “Interventions to improve the appropriate use of polypharmacy in older people” [24] as the key document and our starting point. Further evidence was identified through snowballing. We restricted our searches to systematic reviews.

In total, the search for similar articles detected 123 systematic reviews, with a further 28 identified through snowballing. Bibliographic details on the identified studies were imported into EndNote© and uploaded to Covidence©, and duplicates were removed. Two independent reviewers (M.-S.B. and AI.G.G.) screened the titles, abstracts and full-texts, and included the systematic reviews that fulfilled the inclusion criteria presented in Table 2:

Forty-eight systematic reviews were included in the realist synthesis, most of which concerned interventions to optimize prescribing by, for example, raising awareness of potentially inappropriate medications, using medication reviews, eliciting patient preferences in pharmacotherapy, conducting pharmacist-led interventions, and de-prescribing where appropriate (Supplementary Table S1). The evidence was appraised according to the levels of evidence by the Oxford Centre for Evidence-Based Medicine [25]. In addition to the formal appraisal of evidence levels, the quality of the systematic reviews was assessed using the criteria of the Scottish Intercollegiate Guidelines Network (SIGN) [26]. Of the included studies, most were high quality meta-analyses, systematic reviews of RCTs or RCTs with a very low risk of bias.

### 3.3. Extract and Synthesize Findings

#### 3.3.1. Systematic Guideline Review

**Figure 1:**
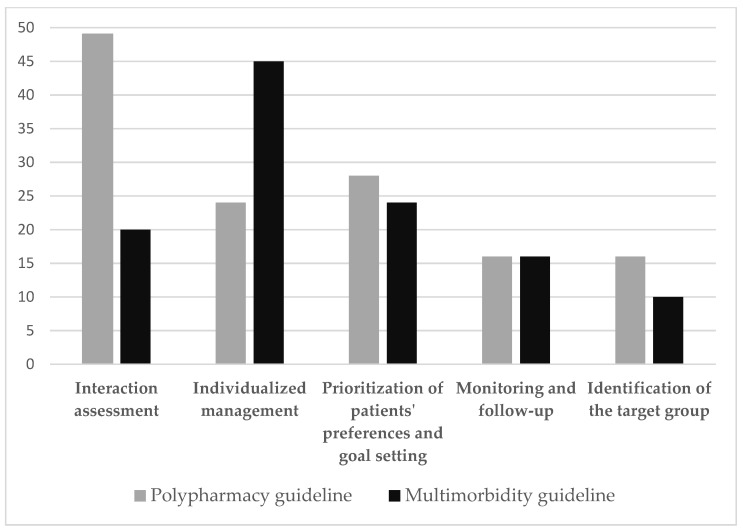
Distribution of recommendations.

For data extraction, we predefined an extraction framework based on the ARIADNE principles, which is a framework to guide care in multimorbid patients [27]. Recommendations from the included guidelines were extracted by five researchers (M.-S.B., J.W.B., T.S.D., A.I.G.G., C.M.) and assigned to the following groups: identification of the target population; interaction assessment; prioritization of patient preferences and agreement on shared treatment goals; individualized management of patients; monitoring and follow-up. Extracted data were reviewed by a further independent researcher and discussed with a third researcher in case of disagreement. Other extracted information included source, year, country, underlying concept/definition of multimorbidity and polypharmacy, target setting, target population, and patient-related outcomes. We synthesized and aggregated the identified recommendations using thematic analysis as advocated in the ARIADNE principles [12]. The distribution of recommendations made in the multimorbidity [14,15,16,17] and the polypharmacy [18,19,20,21] guidelines are presented according to topic in Figure 1.

At a 2-day meeting, the results of the thematic synthesis were discussed and consented with a multidisciplinary international team of 18 experts (geriatrics, primary care, public health and health services research, epidemiology and pharmacy/pharmacology) from seven countries [12]. Group discussions were audio-taped and used for thematic analysis. Triangulation was conducted using the results from the SGR and the group discussion, whereby the outcomes were returned to and then synthesized by participants.

#### 3.3.2. Evidence Searches

Data from the additionally identified systematic reviews were extracted into Excel and assigned to the following categories by one researcher (A.I.G.G.): author; year (reference); context; intervention; outcomes; results; conclusion; guideline chapter; SIGN quality appraisal.

### 3.4. Develop Narrative

#### 3.4.1. Draft of Recommendations and Draft of the Upgraded Guideline

**Figure 2:**
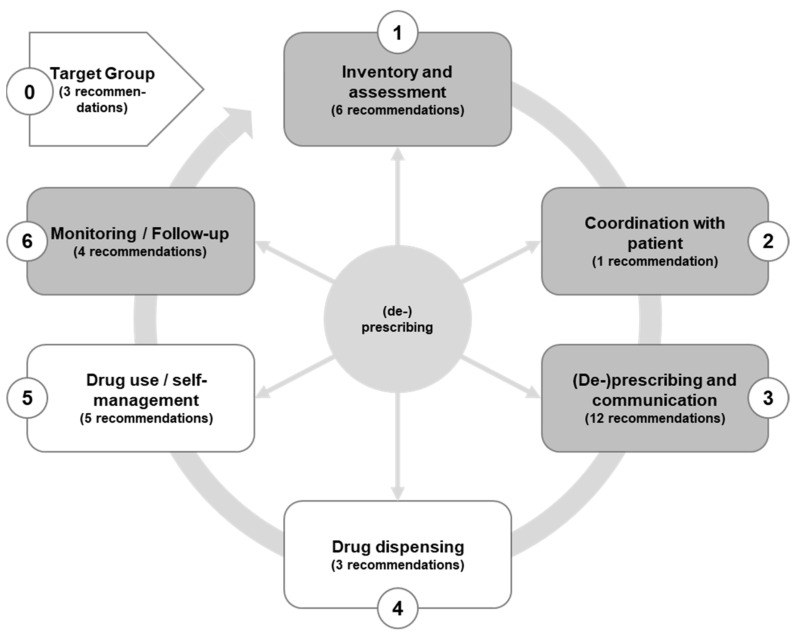
Medication process.

The results of the evidence syntheses were discussed with the LLGH in 24 sessions. The evidence syntheses were presented by M.-S.B., T.S.D., C.M., and the sessions moderated by I.S. Existing chapters were revised and new chapters added, whereby each chapter was broken down into “evidence and rationale” and “practice tips and tools”, based on the results of the syntheses. Additional experts were invited to contribute to specific sections of the guideline by providing information on, for example, the interface between general practice and pharmacies, or de-prescribing. Evidence-based recommendations and statements were formulated with the LLGH. References as well as levels of evidence were added to the recommendations and statements. Since recommendations have a normative character, the formulation of recommendations and statements also took into account their relevance for primary care and the transferability of international evidence to a German health care context. As a result, strong recommendations (e.g., A) could be based on a low level of evidence (e.g., V) and vice versa. The grades of recommendation used in the upgraded guideline are presented in Table 3:

In total, we formulated 34 recommendations and statements, and assigned them to one of the following groups in accordance with the revised framework of the medication process (see Figure 2): identification of target group (*n* = 3), inventory and assessment (*n* = 6), coordination with the patient (*n* = 1), (de-)prescribing and communication (*n* = 12), drug dispensing (*n* = 3), drug use and self-management (*n* = 5), and monitoring and follow-up (*n* = 4).

Steps 1 to 3 include recommendations on medication review as a systematic process. Steps 4 (drug dispensing) and 5 (medication use/self-management) are only indirectly accessible to GPs, and step 6 (monitoring and follow-up) merges with step 1 and reflects the long-term aim of primary care.

In addition to the upgraded long version of the guideline, a short version, as well as a guideline report were developed.

#### 3.4.2. Inter-Professional Consensus Conference

Based on a modified version of the RAND appropriateness method, we discussed and agreed upon recommendations with 13 authorized experts from various medical disciplines including nursing and pharmacy, and a trained patient representative [28].

In a first step, participants received the draft of the upgraded long version of the guideline, including a list of all proposed recommendations and statements. Before discussing them with the other participants, they were required to use a six-point Likert scale to rank them online (1 = strongly disagree, 6 = strongly agree). The results were made available before the consensus conference began and showed that agreement between the participants was high (median: 6; range: 5–6).

In a second step, all 34 recommendations were discussed and agreement was reached at an inter-professional consensus conference. The consensus conference was moderated by an external moderator (C.M.-B.) and was held online due to the COVID-19 pandemic. Recommendations were discussed intensively by participants in the consensus conference and modified live via screen sharing. The discussion was supported by non-voting participants (M.-S.B., T.S.D., I.S., C.M.), who provided evidence and reasoning, documented changes and took notes. Of the 34 recommendations, 31 were modified during the consensus process. Most modifications were minor changes in wording and/or strength of recommendation without changing the content of the recommendations. Some recommendations were further improved after the discussion with participants by adding additional information (e.g., by considering “lifestyle-related factors” during the “interaction assessment process” (recommendation 1-1)). Recommendations were agreed upon live using an anonymous online voting system (approval, rejection, abstention). The level of agreement was 100% for 27 of the recommendations, 92% for 5 recommendations, and 90% and 82% for one recommendation each.

A new version of the guideline draft was then sent to conference participants, who were asked to use the Delphi process to obtain comprehensive feedback on the full text guideline from the organizations they represented. The feedback was discussed by the LLGH and the guideline revised where necessary. To ensure transparency, a point-to-point-reply format was used to document all comments and changes, and it was published along with the guideline report [29].

#### 3.4.3. Feasibility Test

GP practices from the practice-based research network SaxoForN (Saxony and Hesse) and Bielefeld were recruited to test the new guideline for feasibility under field conditions. Twenty-six GPs from 22 practices agreed to participate. Participating GPs received both the long and the short version of the guideline, and they were asked to provide feedback using a 10-item response form that asked for feedback on overall impression, clarity of presentation, definition of the target group, inventory and medication assessment, coordination with patients, providing patients with information on their medication, cooperation with pharmacists, medication use and self-management, monitoring and follow-up, and whether they would recommend the guideline to their colleagues. In addition, GPs were asked to treat around five patients from their practices in accordance with guideline recommendations, and to prepare anonymized case documentation at baseline (the initial consultation) and, if possible, for a follow-up consultation. The participating GPs were asked to complete the questionnaires within a month, either online or paper based.

Of the 26 GPs, 15 from 12 practices responded within the specified time period. They provided structured feedback and documented 67 initial and 27 follow-up consultations with patients. Although 92% of the GPs would recommend the guideline to their colleagues, 58% thought it was too long, and the two-page short version of the guideline was therefore frequently considered preferable. Eighty-three percent of participants thought the target group was clearly defined. While 75% of the participating GPs regarded the recommendations as helpful, only 25% thought important recommendations could be found easily. Recommendations concerning coordination with patients, providing patients with information on their medication, cooperation with pharmacists, medication use and self-management, and monitoring/follow-up were found helpful by 58%, 58%, 75% and 83% of GPs, respectively.

Results from the feasibility test are presented in Table 4.

In 57% of the initial consultations, use of the guideline enabled participants to identify problems, and in 49% of cases, subsequent medication adjustments were made. In 56% of the follow-up consultations, patients reported an improvement in their health. Patients treated by the GPs were between 43 and 92 years old, with 93% of them taking more than 5 long-term medications and 91% having 3 or more chronic diseases. During the initial consultations, GPs reported de-prescribing more than one drug in 46% of patients and prescribing one or more new drugs in 26% of patients. In a further 26% of patients, a change in the dose was made. Seventy-two percent of patients reported obtaining their medications from a particular pharmacy, and 70% said they had an up-to-date medication plan. Forty-six percent of patients felt their treatment was a burden, and GPs tried to reduce this in 28% of cases. At follow-up, 74% of patients had an up-to-date medication plan, and in 59% of cases, medication changes agreed upon during the initial consultation were rated as successful by the GPs. However, in 14.8% of cases, the onset of new nonspecific symptoms/adverse drug reactions was reported.

Feedback was discussed with the LLGH and changes were made and documented where appropriate. The pre-final version of the guideline was again sent to the boards of the societies involved in the consensus conference for final approval and authorization. Finally, the upgraded guideline on polypharmacy was published (short and long version [30,31]) together with a detailed guideline report [29]. The upgraded and updated guideline will be distributed and implemented, e.g., via quality circles and medical networks. Furthermore, it will be part of a new teaching concept for medical and pharmacy students, which aim is to strengthen interprofessional cooperation.

## 4. Discussion

This guideline upgrade was part of the EVITA project, whose aim it is to develop and implement a nationwide structured management program on polypharmacy. Current disease management programs (DMPs) focus on single diseases and do not adequately take patients with multiple chronic conditions into account. The new guideline was therefore developed to serve as a basis for the development and implementation of a structured polypharmacy management program. The realist synthesis identified issues not previously addressed in the guideline (e.g., treatment burden, role of care manager, tools), led to a more stringent framework and provided evidence for the recommendations. The guideline is expected to help GPs and other health care professionals involved in the care of patients with multimorbidity and polypharmacy and can provide evidence-based decision support. Use of a realist synthesis allowed us to ensure the scope of the review was relevant to stakeholders, and helped us to understand the mechanisms of action, and to systematically develop evidence-based recommendations for the guideline. Stakeholders’ perspectives, feasibility and guideline acceptance were assessed and considered throughout the process.

The importance of involving stakeholders in guideline development has already been described by organizations such as the National Institute of Health and Care Excellence (NICE) [32]. Similarly to NICE, a multidisciplinary committee (Guideline Group of General Practitioners of Hesse, pharmacologist, pharmacist, researchers) was involved in the decision-making process and substantially contributed to the refinement and agreement of research questions, the discussion of the evidence, development of recommendations for practice, and the development of implementation strategies. While in the validation and consultation process used by NICE, the draft of a new guideline was posted online and commented on by registered stakeholders [32]. We invited representatives from various medical disciplines to participate in a consensus conference and to discuss their recommendations at a one-day meeting according to the requirements of the AWMF (The Association of the Scientific Medical Societies in Germany) for S3 guidelines. This conference was followed by use of the Delphi technique, which involved multiple consultations with the included medical societies. Furthermore, we initiated a feasibility test of the guideline before its final authorization and publication.

However, because of the broad range of issues covered in the guideline meant it was not feasible to address all the topics identified in the focus groups in depth. By prioritizing issues, the LLGH enabled us to limit the scope of the review questions, but our evidence searches and synthesis may have missed other topics that may have been relevant to address in this context. Another limitation is that recommendations were formulated with an eye to their relevance to primary care and their use in a German health care context, and some strong recommendations were based on a low level of evidence and vice versa. These may not be suitable for use in other health care contexts.

To conclude, the steps of the realist synthesis are suitable for developing a complex guideline. However, prerequisites are a suitable financial framework, time resources and committed guideline developers.

## Figures and Tables

**Table 1:** Stages and methods of the realist synthesis.

Stage	Methods	Aim
Define the scope of the review	1. Focus groups with GPs	To discuss the first version of the polypharmacy guideline, and to identify need for changes and prioritize issues of interest
Search for and appraise the evidence	2. Systematic guideline review [11] 3. Evidence searches	To identify, analyze and synthesize evidence-based recommendations from international guidelines on multimorbidity and polypharmacy, and from systematic reviews
Extract and synthesize findings		
Develop narrative	4. Guideline update by the LLGH and inter-professional consensus conference	To update the guideline, formulate recommendations and achieve consensus among stakeholders/authorized experts on recommendations for the guideline.
	5. Feasibility test	Pilot-test the new guideline under field conditions

GPs—general practitioners; LLGH—Leitliniengruppe Hessen.

**Table 2:** Inclusion and exclusion criteria in the search for additional evidence.

Inclusion Criteria	Exclusion Criteria
Population: patients with multimorbidity and polypharmacy	Setting was not primary care (e.g., inpatients) Patients did not have multimorbidity or polypharmacy
Setting: primary care and nursing homes	
Outcome or phenomenon of interest: - Identification of target population - Tools to support medication review - Tools to assess patients’ preferences and priorities - Interventions to optimize medication use	

**Table 3:** Levels of recommendation.

Level of Recommendation	Syntax
**A** Strong recommendation	Should/should not
**B** Recommendation	Is reasonable/is not reasonable
**O** Weak recommendation	May be considered

**Table 4:** Results from the feasibility test.

Consultation	Item	%
**Initial consultation**	**Identification of problems ^1^:**	56.7
	Drug interactions	32.8
	Other problems	44.8
	**Communication of treatment goals**	88.1
	**Changes in medications ^1^:**	49.3
	Deprescribed ≥ 1 drug	46.2
	Prescribed ≥ 1 drug	26.2
	Dosage change in ≥ 1 drug	26.2
	Communicated safety netting in case of ADR	83.6
	**Patients takes OTC medication:**	
	Yes	34.3
	No	43.3
	Unknown	19.4
	No answer	3.0
	**Patient has up-to-date medication plan**	70.2
	**Patient obtains medications from a particular pharmacy:**	
	Yes	71.6
	No	1.5
	Unknown	26.9
	**Patient reports treatment burden:**	
	Yes	46.3
	No	50.8
	Unknown	3.0
	If yes, interventions to reduce treatment burden:	
	Yes	28.4
	No	11.9
	Not possible	16.4
	No answer	43.3
**Follow-up consultation**	**Patient has up-to-date medication plan**	74.0
	**Medication changes were successful:**	
	Yes	59.3
	No	22.2
	Unchanged	14.8
	No answer	3.7
	**Onset of new ADRs/symptoms:**	
	Yes	14.8
	No	81.5
	Unknown	3.7

^1^ multiple answers possible; ADR—adverse drug reaction; OTC—over the counter.

## Data Availability

Data is contained within the article or Supplementary Materials.

## Supplementary Materials

The following are available online at https://www.mdpi.com/article/10.3390/jpm12010069/s1, Table S1: Bibliographic details of the included systematic reviews.

## Appendix A. EVITA Study Group Members and Affiliation

Maria-Sophie Brückle ^1^, Truc Sophia Dinh ^1^, Ferdinand Michael Gerlach ^1^, Ana Isabel González-González ^1^, Wolfgang Greiner ^2^, Tobias Haber ^3^, Veronika Lappe ^4^, Ursula Marschall ^5^, Christiane Muth ^1,6^, Maximilian Pilz ^7^, Achim Schmidtko ^8^, Ingrid Schubert ^4^, Manfred Schubert-Zsilavecz ^9^, Svenja Seide ^7^, Marjan van den Akker ^1^, Julian Witte ^2^

^1^ Institute of General Practice, Goethe-University Frankfurt, Theodor-Stern-Kai 7, 60590 Frankfurt am Main, Germany; ^2^ Department of Health Economics and Health Care Management, Faculty of Health Science, Bielefeld University, Universitaetsstr. 25, 33615 Bielefeld, Germany; ^3^ INSIGHT Health GmbH & Co. KG, Auf der Lind 10a, 65529 Waldems-Esch, Germany; ^4^ PMV Research Group, Faculty of Medicine and University Hospital Cologne, University of Cologne, Herderstrasse 52, 50931 Cologne, Germany; ^5^ Head of Department Medicine/Health Care Research, Barmer, Lichtscheider Str. 89, 42285 Wuppertal, Germany; ^6^ Department of General Practice and Family Medicine, Medical Faculty OWL, University of Bielefeld, Universitaetsstrasse 25, 33615 Bielefeld, Germany; ^7^ Institute of Medical Biometry and Informatics, Heidelberg University Hospital, Im Neuenheimer Feld 130.3, 69120 Heidelberg, Germany; ^8^ Institute of Pharmacology and Clinical Pharmacy, Goethe University, Max-von-Laue-Str. 9, 60438, Frankfurt am Main, Germany; ^9^ Institute of Pharmaceutical Chemistry/ZAFES, Goethe University, Max-von-Laue-Str. 9, 60438, Frankfurt am Main, Germany.

## References

[1] Violan C., Foguet-Boreu Q., Flores-Mateo G., Salisbury C., Blom J., Freitag M., Glynn L., Muth C., Valderas J.M. (2014). Prevalence, determinants and patterns of multimorbidity in primary care: A systematic review of observational studies. PLoS ONE.

[2] Morin L., Vetrano D.L., Rizzuto D., Calderón-Larrañaga A., Fastbom J., Johnell K. (2017). Choosing Wisely? Measuring the Burden of Medications in Older Adults near the End of Life: Nationwide, Longitudinal Cohort Study. Am. J. Med..

[3] Haefeli W.E., Meid A.D. (2018). Pill-count and the arithmetic of risk: Evidence that polypharmacy is a health status marker rather than a predictive surrogate for the risk of adverse drug events. Int. J. Clin. Pharmacol. Ther..

[4] Thavorn K., Maxwell C.J., Gruneir A., Bronskill S.E., Bai Y., Koné Pefoyo A.J., Petrosyan Y., Wodchis W.P. (2017). Effect of socio-demographic factors on the association between multimorbidity and healthcare costs: A population-based, retrospective cohort study. BMJ Open.

[5] Institute for Quality and Efficacy in Health Care What Are Disease Management Programs (DMPs)?. https://www.ncbi.nlm.nih.gov/books/NBK279412/.

[6] Tinetti M.E., Fried T.R., Boyd C.M. (2012). Designing health care for the most common chronic condition–multimorbidity. JAMA.

[7] Greenhalgh T., Wong G., Westhorp G., Pawson R. (2011). Protocol--realist and meta-narrative evidence synthesis: Evolving standards (RAMESES). BMC Med. Res. Methodol..

[8] Rycroft-Malone J., McCormack B., Hutchinson A.M., DeCorby K., Bucknall T.K., Kent B., Schultz A., Snelgrove-Clarke E., Stetler C.B., Titler M. (2012). Realist synthesis: Illustrating the method for implementation research. Implement. Sci..

[9] Rycroft-Malone J., McCormack B., DeCorby K., Hutchinson A. (2011). Realist synthesis. The Research Process in Nursing.

[10] Pawson R., Greenhalgh T., Harvey G., Walshe K. (2004). Realist Synthesis: An Introduction: RMP Methods Paper 2/2004.

[11] Muth C., Gensichen J., Beyer M., Hutchinson A., Gerlach F.M. (2009). The systematic guideline review: Method, rationale, and test on chronic heart failure. BMC Health Serv. Res..

[12] Muth C., Blom J.W., Smith S.M., Johnell K., Gonzalez-Gonzalez A.I., Nguyen T.S., Brueckle M.-S., Cesari M., Tinetti M.E., Valderas J.M. (2019). Evidence supporting the best clinical management of patients with multimorbidity and polypharmacy: A systematic guideline review and expert consensus. J. Intern. Med..

[13] AWMF AWMF-Regelwerk Leitlinien: Stufenklassifikation Nach Systematik. https://www.awmf.org/leitlinien/awmf-regelwerk/ll-entwicklung/awmf-regelwerk-01-planung-und-organisation/po-stufenklassifikation.html.

[14] Scherer M., Wagner H.-O., Lühmann D.E.A. Multimorbidität S3-Leitlinie: AWMF-Register-Nr. 053-047. DEGAM-Leitlinie Nr. 20. https://www.awmf.org/uploads/tx_szleitlinien/053-047l_S3_Multimorbiditaet_2018-01.pdf.

[15] NICE Older People with Social Care Needs and Multiple Long-Term Conditions. https://www.nice.org.uk/guidance/ng22.

[16] NICE Multimorbidity: Clinical Assessment and Management. Multimorbidity: Assessment, Prioritisation and Management of Care for Older People with Commonly Occuring Multimorbidity. https://www.nice.org.uk/guidance/ng56.

[17] AGS (2012). Guiding principles for the care of older adults with multimorbidity: An approach for clinicians: American Geriatrics Society Expert Panel on the Care of Older Adults with Multimorbidity. J. Am. Geriatr. Soc..

[18] NICE Medicines Optimisation: The Safe and Effective use of Medicines to Enable the Best Possible Outcomes. https://www.nice.org.uk/guidance/ng5/evidence/full-guideline-pdf-6775454.

[19] NHG Multidisciplinaire Richtlijn Polyfarmacie Bij Ouderen. https://www.nhg.org/sites/default/files/content/nhg_org/uploads/polyfarmacie_bij_ouderen.pdf.

[20] Bergert F.W., Braun M., Ehrenthal K., Feßler J., Gross J., Hüttner U., Kluthe B., Liesenfeld A., Seffrin J., Vetter G. (2014). Recommendations for treating adult and geriatric patients on multimedication. Int. J. Clin. Pharmacol. Ther..

[21] Peralta-Pedrero M.L., Valdivia-Ibarra F.J., Hernandez-Manzano M.E.A. (2013). Clinical practice guideline. Drug prescription in elderly. Rev. Med. Inst. Mex. Seguro Soc..

[22] Semlitsch T., Jeitler K., Kopp I.B., Siebenhofer A. (2014). Entwicklung einer praktikablen Mini-Checkliste zur Bewertung der methodischen Leitlinienqualität. Z. Evid. Fortbild. Qual. Gesundhwes..

[23] Semlitsch T., Blank W.A., Kopp I.B., Siering U., Siebenhofer A. (2015). Evaluating Guidelines: A Review of Key Quality Criteria. Dtsch. Arztebl. Int..

[24] Rankin A., Cadogan C.A., Patterson S.M., Kerse N., Cardwell C.R., Bradley M.C., Ryan C., Hughes C. (2018). Interventions to improve the appropriate use of polypharmacy for older people. Cochrane Database Syst. Rev..

[25] Oxford Centre for Evidence-Based Medicine Levels of Evidence. https://www.cebm.ox.ac.uk/resources/levels-of-evidence/oxford-centre-for-evidence-based-medicine-levels-of-evidence-march-2009.

[26] Scottish Intercollegiate Guidelines Network Risk Estimation and the Prevention of Cardiovascular Disease. http://www.sign.ac.uk.

[27] Muth C., van den Akker M., Blom J.W., Mallen C.D., Rochon J., Schellevis F.G., Becker A., Beyer M., Gensichen J., Kirchner H. (2014). The Ariadne principles: How to handle multimorbidity in primary care consultations. BMC Med..

[28] Fitch K., Bernstein S.I., Aguilar M., Burnand B., LaCalle J.R., Lazaro P., van het Loo M., McDonnell J., Vader J., Kahan J.P. He RAND/UCLA Appropriateness Method User’s Manual. https://www.rand.org/pubs/monograph_reports/MR1269.html.

[29] Leitliniengruppe Hessen D. S3-Leitlinie Multimedikation, Leitlinienreport. https://www.awmf.org/uploads/tx_szleitlinien/053-043m_S3_Multimedikation_2021-07.pdf.

[30] Leitliniengruppe Hessen D. S3-Leitlinie Multimedikation, Kurzfassung. https://www.awmf.org/uploads/tx_szleitlinien/053-043k_S3_Multimedikation_2021-07.pdf.

[31] Leitliniengruppe Hessen D. S3-Leitlinie Multimedikation, Langfassung: AWMF-Registernummer: 053-043. https://www.awmf.org/uploads/tx_szleitlinien/053-043l_S3_Multimedikation_2021-08.pdf.

[32] NICE Developing NICE Guidelines: The Manual: Process and Methods [PMG20]. https://www.nice.org.uk/process/pmg20/chapter/reviewing-research-evidence.

