# Supplementary material for: Evidence-Based Decision Support for a Structured Care Program on Polypharmacy in Multimorbidity: A Guideline Upgrade Based on a Realist Synthesis
Source: J Pers Med. 2022 Jan 7;12(1):69. doi: 10.3390/jpm12010069 (PMC8778077; doi:10.3390/jpm12010069)
Supplement: Supplementary file 1 [file jpm-12-00069-s001.zip › jpm-1508055-supplementary.pdf]

Table S1. List of included studies

1. Alldred DP, Kennedy M-C, Hughes C, Chen TF, Miller P. Interventions to optimise prescribing for older people in care homes. Cochrane Database Syst Rev [Internet]. 2016 Feb 12; Available from: http://doi.wiley.com/10.1002/14651858.CD009095.pub3
2. Anderson LJ, Schnipper JL, Nuckols TK, Shane R, Sarkisian C, Le MM, et al. A systematic overview of systematic reviews evaluating interventions addressing polypharmacy. Am J Heal Pharm. 2019;76(21):1777–87.
3. Anderson K, Stowasser D, Freeman C, Scott I. Prescriber barriers and enablers to minimising potentially inappropriate medications in adults: A systematic review and thematic synthesis. BMJ Open. 2014;4(12).
4. de Barra M, Scott CL, Scott NW, Johnston M, de Bruin M, Nkansah N, et al. Pharmacist services for non-hospitalised patients. Cochrane Database Syst Rev [Internet]. 2018 Sep 4; Available from: http://doi.wiley.com/10.1002/14651858.CD013102
5. Cameron ID, Dyer SM, Panagoda CE, Murray GR, Hill KD, Cumming RG, et al. Interventions for preventing falls in older people in care facilities and hospitals. Cochrane Database Syst Rev [Internet]. 2018 Sep 7; Available from: http://doi.wiley.com/10.1002/14651858.CD005465.pub4
6. Christensen M, Lundh A. Medication review in hospitalised patients to reduce morbidity and mortality. Cochrane Database Syst Rev [Internet]. 2016 Feb 20; Available from: http://doi.wiley.com/10.1002/14651858.CD008986.pub3
7. Eidam A, Roth A, Lacroix A, Goisser S, Seidling HM, Haefeli WE, et al. Methods to assess patient preferences in old age pharmacotherapy – A systematic review. Patient Prefer Adherence. 2020;14:467–97.
8. Ellis G, Gardner M, Tsiachristas A, Langhorne P, Burke O, Harwood RH, et al. Comprehensive geriatric assessment for older adults admitted to hospital. Cochrane Database Syst Rev. 2017;2017(9).
9. Geurts MME, Talsma J, Brouwers JRBJ, de Gier JJ. Medication review and reconciliation with cooperation between pharmacist and general practitioner and the benefit for the patient: a systematic review. Br J Clin Pharmacol. 2012 Jul;74(1):16–33.
10. Gillaizeau F, Chan E, Trinquart L, Colombet I, Walton R, Rège-Walther M, et al. Computerized advice on drug dosage to improve prescribing practice. Cochrane Database Syst Rev [Internet]. 2013 Nov 12; Available from: http://doi.wiley.com/10.1002/14651858.CD002894.pub3
11. Gillespie LD, Robertson MC, Gillespie WJ, Sherrington C, Gates S, Clemson LM, et al. Interventions for preventing falls in older people living in the community. Cochrane Database Syst Rev [Internet]. 2012 Sep 12; Available from: http://doi.wiley.com/10.1002/14651858.CD007146.pub3
12. Guirguis-Blake J, Michael Y, Perdue L, Coppola E, Beil T, Thompson J. Interventions to Prevent Falls in Community-Dwelling Older Adults: A Systematic Review for the U.S. Preventive Services Task Force. Evid Synth No 159 [Internet]. 2018;AHRQ Publi(159):Rockville, MD: Agency for HEalthcare Research and. Available from: https://www.ncbi.nlm.nih.gov/pubmed/30234932
13. Hill-Taylor B, Sketris I, Hayden J, Byrne S, O'Sullivan D, Christie R. Application of the STOPP/START criteria: a systematic review of the prevalence of potentially inappropriate prescribing in older adults, and evidence of clinical, humanistic and economic impact. J Clin Pharm Ther [Internet]. 2013 Oct;38(5):360–72. Available from: http://doi.wiley.com/10.1111/jcpt.12059
14. Holland R, Desborough J, Goodyer L, Hall S, Wright D, Loke YK. Does pharmacist-led medication review help to reduce hospital admissions and deaths in older people? A systematic review and meta-analysis. Br J Clin Pharmacol. 2008 Mar;65(3):303–16.
15. Hopewell S, Adedire O, Copsey B, Boniface G, Sherrington C, Clemson L, et al. Multifactorial and multiple component interventions for preventing falls in older people living in the community (Review). Cochrane Database Syst Rev. 2018;(7):CD012221.

16. Huiskes VJB, Burger DM, van den Ende CHM, van den Bemt BJF. Effectiveness of medication review: a systematic review and meta-analysis of randomized controlled trials. BMC Fam Pract [Internet]. 2017;18(5). Available from: http://link.springer.com/10.1023/A:1015570104121

17. Iyer S, Naganathan V, McLachlan AJ, Le Couteur DG. Medication withdrawal trials in people aged 65 years and older: A systematic review. Drugs and Aging. 2008;25(12):1021–31.

18. Johansson T, Abuzahra ME, Keller S, Mann E, Faller B, Sommerauer C, et al. Impact of strategies to reduce polypharmacy on clinically relevant endpoints: a systematic review and meta-analysis. Br J Clin Pharmacol [Internet]. 2016 Aug;82(2):532–48. Available from: http://doi.wiley.com/10.1111/bcp.12959

19. Jokanovic N, Tan ECK, van den Bosch D, Kirkpatrick CM, Dooley MJ, Bell JS. Clinical medication review in Australia: A systematic review. Res Soc Adm Pharm. 2016;12(3):384–418.

20. Katsimpris A, Linseisen J, Meisinger C, Volaklis K. The Association Between Polypharmacy and Physical Function in Older Adults: a Systematic Review. J Gen Intern Med [Internet]. 2019 Sep 25;34(9):1865–73. Available from: http://link.springer.com/10.1007/s11606-019-05106-3

21. Kaur S, Mitchell G, Vitetta L, Roberts MS. Interventions that can Reduce Inappropriate Prescribing in the Elderly. Drugs Aging [Internet]. 2009 Dec;26(12):1013–28. Available from: http://link.springer.com/10.2165/11318890-000000000-00000

22. Khalil H, Bell B, Chambers H, Sheikh A, Avery AJ. Professional, structural and organisational interventions in primary care for reducing medication errors. Cochrane Database Syst Rev [Internet]. 2017 Oct 4; Available from: http://doi.wiley.com/10.1002/14651858.CD003942.pub3

23. Loganathan M, Singh S, Franklin BD, Bottle A, Majeed A. Interventions to optimise prescribing in care homes: systematic review. Age Ageing [Internet]. 2011 Mar 1;40(2):150–62. Available from: http://academic.oup.com/ageing/article/40/2/150/46685

24. Mangin D, Stephen G, Bismah V, Risdon C. Making patient values visible in healthcare: a systematic review of tools to assess patient treatment priorities and preferences in the context of multimorbidity. BMJ Open. 2016 Jun 10;6(6):e010903.

25. Masnoon N, Shakib S, Kalisch-Ellett L, Caughey GE. Tools for Assessment of the Appropriateness of Prescribing and Association with Patient-Related Outcomes: A Systematic Review. Drugs Aging. 2018 Jan 19;35(1):43–60.

26. Meid AD, Quinzler R, Freigofas J, Saum K, Schöttker B, Holleczek B, et al. Medication Underuse in Aging Outpatients with Cardiovascular Disease: Prevalence, Determinants, and Outcomes in a Prospective Cohort Study. Reddy H, editor. PLoS One. 2015 Aug 19;10(8):e0136339.

27. Michiels-Corsten M, Gerlach N, Schleef T, Junius-Walker U, Donner-Banzhoff N, Viniol A. Generic instruments for drug discontinuation in primary care: A systematic review. Br J Clin Pharmacol. 2020;86(7):1251–66.

28. Moghadam ST, Velayati F, Sadoughi F, Ehsanzadeh S, Poursharif S. The effects of clinical decision support system for prescribing medication on patient outcomes and physician practice performance: A systematic review and meta-analysis. BMC Med Informatics Decis Mak [Internet]. 2020;00:1–42. Available from: https://doi.org/10.21203/rs.3.rs-18677/v2

29. Monteiro L, Maricoto T, Solha I, Ribeiro-Vaz I, Martins C, Monteiro-Soares M. Reducing Potentially Inappropriate Prescriptions for Older Patients Using Computerized Decision Support Tools: Systematic Review. J Med Internet Res. 2019 Nov 14;21(11):e15385.

30. Patton DE, Hughes CM, Cadogan CA, Ryan CA. Theory-Based Interventions to Improve Medication Adherence in Older Adults Prescribed Polypharmacy: A Systematic Review. Drugs and Aging. 2017;34(2):97–113.

31. Page AT, Clifford RM, Potter K, Schwartz D, Etherton-Beer CD. The feasibility and effect of deprescribing in older adults on mortality and health: a systematic review and meta-analysis. Br J Clin Pharmacol. 2016;583–623.

32. Pazan F, Kather J, Wehling M. A systematic review and novel classification of listing tools to improve medication in older people. Eur J Clin Pharmacol. 2019 May;75(5):619–25.

33. Rankin A, Cadogan CA, Patterson SM, Kerse N, Cardwell CR, Bradley MC, et al. Interventions to improve the appropriate use of polypharmacy for older people. Cochrane Database Syst Rev. 2018 Sep 3;9.

34. Redmond P, Grimes TC, McDonnell R, Boland F, Hughes C, Fahey T. Impact of medication reconciliation for improving transitions of care. Cochrane Database Syst Rev [Internet]. 2018 Aug 23; Available from: http://doi.wiley.com/10.1002/14651858.CD010791.pub2

35. Reeve E, To J, Hendrix I, Shakib S, Roberts MS, Wiese MD. Patient barriers to and enablers of deprescribing: A systematic review. Drugs and Aging. 2013;30(10):793–807.

36. Royal S, Smeaton L, Avery AJ, Hurwitz B, Sheikh A. Interventions in primary care to reduce medication related adverse events and hospital admissions: Systematic review and meta-analysis. Qual Saf Heal Care. 2006;15(1):23–31.

37. Ryan R, Santesso N, Lowe D, Hill S, Grimshaw JM, Prictor M, et al. Interventions to improve safe and effective medicines use by consumers: an overview of systematic reviews. Cochrane Database Syst Rev [Internet]. 2014 Apr 29; Available from: http://doi.wiley.com/10.1002/14651858.CD007768.pub3

38. Seppala LJ, Wermelink AMAT, de Vries M, Ploegmakers KJ, van de Glind EMM, Daams JG, et al. Fall-Risk-Increasing Drugs: A Systematic Review and Meta-Analysis: II. Psychotropics. J Am Med Dir Assoc. 2018;19(4):371.e11-371.e17.

39. Seppala LJ, van de Glind EMM, Daams JG, Ploegmakers KJ, de Vries M, Wermelink AMAT, et al. Fall-Risk-Increasing Drugs: A Systematic Review and Meta-analysis: III. Others. J Am Med Dir Assoc. 2018;19(4):372.e1-372.e8.

40. Sheehan OC, Leff B, Ritchie CS, Garrigues SK, Li L, Saliba D, et al. A systematic literature review of the assessment of treatment burden experienced by patients and their caregivers. BMC Geriatr. 2019;19(1):1–11.

41. Smith SM, Wallace E, O'Dowd T, Fortin M. Interventions for improving outcomes in patients with multimorbidity in primary care and community settings. Cochrane Database Syst Rev [Internet]. 2016 Mar 15; Available from: http://doi.wiley.com/10.1002/14651858.CD006560.pub3

42. Stevenson FA, Cox K, Britten N, Dundar Y. A systematic review of the research on communication between patients and health care professionals about medicines: the consequences for concordance. Heal Expect. 2004 Aug 20;7(3):235–45.

43. Tecklenborg S, Byrne C, Cahir C, Brown L, Bennett K. Interventions to Reduce Adverse Drug Event-Related Outcomes in Older Adults: A Systematic Review and Meta-analysis. Drugs Aging [Internet]. 2020 Feb 10;37(2):91–8. Available from: http://link.springer.com/10.1007/s40266-019-00738-w

44. Thomas R, Huntley AL, Mann M, Huws D, Elwyn G, Paranjothy S, et al. Pharmacist-led interventions to reduce unplanned admissions for older people: A systematic review and meta-analysis of randomised controlled trials. Age Ageing. 2014;43(2):174–87.

45. Verloo H, Chiolero A, Kiszio B, Kampel T, Santschi V. Nurse interventions to improve medication adherence among discharged older adults: A systematic review. Age Ageing. 2017;46(5):747–54.

46. Viswanathan M, Kahwati LC, Golin CE, Blalock SJ, Coker-Schwimmer E, Posey R, et al. Medication therapy management interventions in outpatient settings: A systematic review and meta-analysis. JAMA Intern Med. 2015;175(1):76–87.

47. Weeks G, George J, Maclure K, Stewart D. Non-medical prescribing versus medical prescribing for acute and chronic disease management in primary and secondary care. Cochrane Database Syst Rev. 2016;2016(11).

48. Welsh TJ, van der Wardt V, Ojo G, Gordon AL, Gladman JRF. Anticholinergic Drug Burden Tools/Scales and Adverse Outcomes in Different Clinical Settings: A Systematic Review of Reviews. Drugs and Aging [Internet]. 2018;35(6):523–38. Available from: https://doi.org/10.1007/s40266-018-0549-z
